# What Difference Does a Visit Make? Changes in Animal Welfare Perceptions after Interested Citizens Tour a Dairy Farm

**DOI:** 10.1371/journal.pone.0154733

**Published:** 2016-05-31

**Authors:** Beth Ann Ventura, Marina A. G. von Keyserlingk, Hannah Wittman, Daniel M. Weary

**Affiliations:** 1 Animal Welfare Program, University of British Columbia, Vancouver, BC, Canada; 2 Centre for Sustainable Food Systems, University of British Columbia, Vancouver, BC, Canada; ETH Zurich, SWITZERLAND

## Abstract

Citizens’ concerns about farm animal welfare are often dismissed on the assumption that they are not well informed about farming practices. We conducted exploratory surveys of interested citizens (n = 50) before and after a self-guided tour of a 500-head dairy farm. ‘Before’ survey questions explored perceptions, concerns, and values about dairy cattle farming and welfare, in addition to a short knowledge-based quiz on dairy cattle husbandry. An ‘after’ survey explored the extent to which these constructs shifted after the tour. Before, most participants correctly answered quiz questions about general feeding and housing practices, but scores were low on questions about specific practices such as cow-calf separation. Participants considered several elements as necessary for a ‘good’ life for dairy cattle: fresh food and water, pasture access, gentle handling, space, shelter, hygiene, fresh air and sunshine, social companions, absence of stress, health, and safety from predators. These elements reflect a diverse conception of animal welfare that incorporates values for physical and mental well-being, natural living, and humane care. The visit had a mixed effect on perceptions of whether dairy cows had a ‘good’ life, improving perceptions for a quarter of participants, worsening perceptions in a third, with no shift in the remaining participants. The visit appeared to mitigate some concerns (e.g., provision of adequate food and water, gentle humane care) while reinforcing or eliciting others (e.g., lack of pasture access, early cow-calf separation). Moreover, animal welfare-relevant values held by participants (e.g., natural living, care) appeared to play an important role in influencing perceptions of farm practices. These results suggest that education and exposure to livestock farming may resolve certain concerns, but other concerns will likely persist, especially when practices conflict with deeply held values around animal care.

## Introduction

Animal agriculture has come under increasing criticism with respect to farm animal welfare, but these critiques are sometimes dismissed by people working in agriculture on the basis that public concerns are misinformed [1–3]. The idea that public criticisms are based on misunderstandings of the true nature of specialized practices is known as the knowledge deficit model of public understanding. One implication of this model, also referred to as the knowledge gap, informational deficit, or cognitive deficit [4,5], is that concerns can be corrected through education to bring public opinions into line with those of experts (see [6] for a detailed exploration of the assumptions and limitations of the knowledge deficit model).

In addition to differences in knowledge between people within and outside the livestock industries, people may differ in their values [6,7]. Values are “*desirable*, *trans-situational goals…that serve as guiding principles in people’s lives*,” [8], and as such are fundamental in both belief and attitude formation [9,10]. With respect to agricultural systems and animal welfare, values also function as criteria that people use to evaluate methods of production [11,12]. Concerns are then elicited when an individual encounters something in conflict with their values. Since both knowledge and values contribute to concerns [13], it is important that both be incorporated into research seeking to understand public concern about farm animal welfare.

European research indicates that many citizens do indeed lack knowledge of, and experience with, livestock production [12,14,15], but there has been little relevant work to date on North Americans. Similarly, the Welfare Quality^®^ projects have begun to examine values relevant to farm animal welfare among European citizens, but except for market surveys [16,17] and quantitative surveys on consumer preferences [18], few North American studies have examined what citizens know and value about farm animal welfare [19–21]. In light of concerns that citizens are ignorant of livestock farming, there have been calls for studies based on real-life experience, for example, once citizens have been introduced to farm life in person. Except for two Dutch studies with dairy cattle and pigs [12,22], respectively, we are not aware of any research that has exposed non-farming citizens to operating farms and gauged their responses.

The aims of the current study were first to describe existing animal welfare perceptions, concerns and values within a group of interested lay citizens before their visit to a working dairy farm, and secondly, to determine how their concerns and values, together with performance on a knowledge-based quiz, shifted after visiting the farm. We focused on dairy because this industry has thus far received less attention in relation to animal welfare than some of the other animal industries [15,23,24].

To provide a strong, positive test of the knowledge deficit hypothesis we recruited participants who were engaged in issues around food, but who were not affiliated with the dairy industry, and we selected a farm that was recognized by the dairy industry as achieving high welfare and production standards [25]. We predicted that performance on the quiz, and participant perceptions and concerns, would shift after touring the farm, but that animal welfare-relevant values, as relatively stable constructs, would not shift.

## Methods

We conducted surveys before and after members of the non-dairy farming public self-toured a working dairy farm in summer 2014 in the Fraser Valley of British Columbia. The University of British Columbia Dairy Research and Education Centre (hereafter ‘the farm’) operates as both a working dairy farm and a research site for the university. The herd consists of approximately 500 Holstein cattle, around 230 of which are milking at any time. Included on the tour were the calf barn, in which participants observed calves housed in individual stalls as well as in small groups, and the main barn, referred to as a freestall, where cows were housed in groups of 12 to 24 in pens where they were able to move around freely. The pens contained one lying stall (cubicle) per cow and a minimum of 60 cm of feed bunk space per cow. Cows were provided free access to a mixed diet consisting of forages and concentrates formulated to meet the needs of cattle for their stage of development and production. These conditions follow the standards required by Canada’s National Code of Practice for the Care and Handling of Dairy Cattle [26]. Pasture was available adjacent to the barns, but cows were housed indoors during the tour. This project received research ethics board approval from the University of British Columbia under certificate number: H14-01689.

### Survey description

The study took place in August 2014 during the annual Slow Food^®^ bicycle tour, during which members of the public toured various crop and livestock farms along a predetermined route. Visitors were invited to participate in a short survey before and after visiting the farm. Before touring the farm, participants completed a 5 to 10 minute survey (‘before’ survey, see Table 1) incorporating both quantitative and qualitative questions. Questions were structured to gauge participants’ baseline perceptions (both general [i.e. top of mind associations] and specific [i.e. assessment of whether cattle have good or poor quality lives]), concerns and values relating to dairy cattle welfare. Because the ‘before’ survey took place before participants toured the farm, these questions all pertained to dairy cattle *in general*. As animal welfare tends to be an emotive topic, the survey was designed to avoid leading questions. For example, the term ‘animal welfare’ was not used to prevent participant bias. Question valence was also balanced through the survey, for example through inclusion of questions about the “good life” (positive) vs. questions about concerns (negative).

**Table 1:** Overview of farm tour survey^1^ on citizen perceptions, values, concerns and knowledge relative to dairy cattle welfare.

Construct	‘Before’	‘After’
Perceptions	*General perceptions*: *(Free association) Write up to five (5) words that come to mind when you think of dairy farming*. (QL^2^)	*Now that you’ve toured the farm*, *write up to five (5) words that come to mind when you think about dairy farming*. (QL)
	*Animal welfare-specific perceptions*: *How confident are you that dairy cows generally have a good life*^2^? (QT)	*What*, *if anything*, *surprised you about the way animals are cared for on this farm*? (QL)
		*Fill in the blank*: *In my opinion*, *the animals on this farm have (better*, *about the same*, *worse*, *or unsure) lives than animals on other dairy farms in British Columbia*. *(QT)*
Values	*In your opinion*, *what does a dairy cow need in order to have a good life*? (QL)	*Do you feel that animals on this farm have a good life*? *Why or why not*? (QL)
Concerns^3^	*What (if any) concerns do you have regarding the quality of life for dairy cattle*? (QL)	*Now that you’ve toured this farm*, *please share any concerns about the quality of life for dairy cattle*, *in general or on this farm*. (QL)
	*Please rank up to three (3) of your top concerns*, *and indicate why they concern you*. (QL)	
Quiz^4^	*A dairy cow needs to have a calf to keep producing milk*. *(True/False)*	All questions asked again.
	*Dairy cows in British Columbia are routinely tied in their stall in the barn*. *(True/False)*	
	*All dairy cows in British Columbia are allowed access to pasture*. *(True/False)*	
	*How many days after birth does the dairy calf typically stay with its mom*? *A) 0 days B) 1 week C) 1 month D) never separated*	
	*Which best describes what most adult cows are typically fed on dairy farms*? *A) milk B) grass C) pre-mixed feed*	

^1^ The ‘before’ survey was framed to elicit responses regarding dairy farming in general, as participants completed the survey upon arrival at the farm and before they entered the dairy barn. Once the participants had completed their tour of the farm they completed the ‘after’ survey. These questions were all worded to elicit responses specific to the farm they had just toured.

^2^ QL = open-ended qualitative response, QT = Quantitative, Likert-scale response.

^3^ As a reflection of attitudes, concerns shed light on underlying values.

^4^ Quiz questions were asked twice, on both the Before and After surveys. Quiz question order was randomized.

Participants also completed a five-question quiz (‘before’ quiz). As the tour took place in the province of British Columbia (Canada), these questions were meant to capture common practices in this province relevant to animal welfare debates and around which there are common misconceptions. As such, we did not consider the answers to reflect a complete depiction of all possible knowledge relevant to dairy cattle welfare.

Participants then embarked on a self-guided tour through the farm. The tour included eight stations positioned throughout the main animal facilities. These stations addressed calf management and housing, Canadian guidelines for on-farm animal care, a day in the life of the dairy cow, and cow health, feeding, reproduction and general behavior. Graduate students staffed each station and were available to answer questions. Participants were free to visit any stations they wanted and were later asked to indicate which stations they had visited. There was no time limit to the tour. Upon completion of the tour, participants completed a 5 to 10 minute ‘after’ survey designed to capture shifts in perceptions, concerns and values. The ‘after’ questions focused on participants’ reactions to this specific farm. Participants were also asked to again answer the same quiz questions presented before they toured the farm (referred to as the ‘after’ quiz).

### Participant sample

Recruitment of participants from a Slow Food^®^-sponsored activity was intentional, as people with high levels of engagement in food and agriculture issues tend to be early participants in, and sometimes disproportionately shape, discourse on contentious issues. We were aware that these individuals would likely be more interested in and knowledgeable about livestock farming than other lay (non-dairy farming) citizens in the population.

A total of 50 participants completed both ‘before’ and ‘after’ surveys and were included in the analysis (see Table 2 for participant demographics). Of these, 30 participants were female, 27 people were between the ages of 35 and 54 with an additional 30% above the age of 55, most (n = 30) had a bachelor’s degree or higher, and the majority had lived most of their lives in urban or suburban settings (n = 40). Of the four people who indicated that they had grown up on a farm, none indicated that they had lived or worked on a dairy farm. Half of the participants indicated that they were not knowledgeable about dairy farming, with an additional 22 people indicating that they were somewhat knowledgeable. All participants lived in Canada at the time of the survey and all but two consumed dairy products; those who did not consume dairy indicated that they were lactose-intolerant.

**Table 2:** Description of participants who completed both 'before' and 'after' surveys for the dairy farm visit (n = 50).

Variable	n
Sex	
*Female*	30
*Male*	20
Age	
*19–34*	7
*35–54*	27
*>55*	15
*Prefer not to say*	1
Country of residence	
*Canada*	50
Where have you lived most of your life?	
*Urban*	22
*Suburban*	18
*Rural*, *not on a farm*	6
*Rural*, *on a farm*^1^	4
Education level	
*Vocational/apprenticeship*	4
*High school diploma*	14
*Undergraduate degree*	12
*Graduate degree*	12
*Professional (e*.*g*. *MD*, *DVM)*	07
*Other*	1
Do you consume dairy?	
*Yes*	48
*No*	2
Knowledge of dairy farming?	
*Very knowledgeable*	3
*Somewhat knowledgeable*	22
*Not knowledgeable*	25
Confidence that dairy cattle generally have a good life?	
*Confident or very confident*	21
*Neutral or unsure*	15
*Somewhat or not confident*	14

^1^ Farms other than dairy cattle farms.

### Analyses

#### Qualitative analysis

Content analysis was used for the qualitative responses [27]. This process involves a thorough reading and re-reading of the generated text, with the researcher(s) noting emerging patterns and assigning themes and sub-themes to related sections of text. Comments were read with the goal of identifying perceptions, concerns and values with respect to the dairy industry and animal welfare. The lead author (B.A. Ventura) coded the data, with an additional researcher trained in qualitative analysis independently coding a subsection of the data to strengthen the robustness of the codes. Agreement between initial coding lists was very high; codes were then discussed until mutually agreed upon schemes were reached, resulting in fully consistent coding by the two coders.

Two main schemes were developed to describe participants’ baseline perceptions and values before the farm visit: 1) ‘industry perceptions’ to describe general perceptions of the dairy industry and 2) ‘FAW values’ to describe values around farm animal welfare (FAW), including what participants valued as part of a good life for dairy cows and their resulting concerns. The ‘industry perceptions’ scheme was derived largely from participants’ free association (i.e. general question about dairy farming, see Table 1) responses.

As part of the coding process for FAW values, Fraser et al.’s [7] description of animal welfare in terms of biological functioning (e.g. physical condition and health), natural living (the degree to which an animal can live a natural life), and affective states (how an animal feels) was used as a starting point to organize comments, but the final coding scheme was expanded beyond this framework based on participants’ responses.

Particularly demonstrative responses are quoted below to illustrate the themes, followed by participant number in brackets (e.g. [P23] to designate Participant #23).

#### Quantitative analysis

Although this was a primarily qualitative study and so not designed to predict effects of measured variables on before- and after-visit responses, we did check for the relationship between the perception shift upon visiting the farm and demographics (sex, age, education level, rural/urban status, self-reported knowledge, and ‘before’ confidence in cattle welfare), the ‘before’ quiz score (out of 5), ‘before’ confidence, and the FAW value expression and range. The relationship between self-reported knowledge and ‘before’ quiz scores was also assessed (see Table 3 for explanation of variables).

**Table 3:** Description, type and levels of demographic and response variables included in analysis of citizen responses before and after visiting the dairy farm.

Variable	Description	Type	Variable levels
Sex	Demographic	Categorical	*Female* or *Male*
Age (yrs)	Demographic	Continuous	*19–24*, *25–34*, *35–44*, *45–54*, *55–64*, or *65+*
Education	Demographic	Categorical	*Vocational/apprenticeship*, *High school diploma*, *undergraduate degree*, *graduate degree*, *professional*, or *other*
Rural/urban status	Demographic	Categorical	*Rural* or *urban*: where *rural = (rural not on a farm* + *rural on a farm)* and *urban* = (*urban* + *suburban)* responses.
Self-reported knowledge	Subjective self-assessment of general knowledge of dairy husbandry	Categorical	*No knowledge*, *At least some knowledge*: Only three people self-reported as *very knowledgeable* so these were re-categorized as *At least some knowledge*
‘Before’ quiz score	Objective score on the ‘before’ quiz on dairy husbandry	Continuous	*0*, *1*, *2*, *3*, 4 or *5* (out of a total of 5 possible) correct responses. Blank responses were incorrect.
‘Before’ confidence	‘Before’ visit confidence about how good of a life dairy cattle have	Continuous	*Confident*, *neutral*, *not confident*: Created by collapsing five to three levels for analysis as we were primarily interested in valence of confidence.
FAW value expression	‘Before’ response: was each animal welfare value criterion as determined from the qualitative analysis expressed?	Categorical	*Yes* or *No*: for each of *biological functioning*, *natural living*, *affective states*, *humane care*, *drugs* and *respect for life*.
FAW value range	‘Before’ response: number of FAW value criteria referenced	Continuous	*0*, *1*, *2*, *3*, *4*, *5*, or *6* values expressed
Perception shift	‘After’ response: Shift in individuals’ perception of the level of animal welfare after farm visit	Continuous	*Positive shift*, *no*, or *negative shift*: Created by comparing ‘before’ confidence against ‘after’ responses on whether cows had a good life. *Positive shift* = improved view of FAW after visit, *no* = no change, *negative shift* = worsened view of FAW. This approach was designed to mitigate repetition and participant drop-out.

We used χ^2^ tests to test for relationships between categorical variables, Spearman rank correlation to test for relationships between two continuous variables, and Kruskal-Wallis tests for relationships between categorical and continuous variables. To test if changes in responses to the quiz questions differed from chance, we compared the direction of the change relative to chance expectations using a binomial test. For example, for the true false questions, the null hypothesis was that as many participants would switch from true to false as vice versa. Alpha was set at 0.05 for all tests. Unless otherwise stated, relationships between variables were not significant.

## Results

### Responses before visiting the dairy farm

#### General perceptions of the dairy industry

Both positive and negative perceptions of the dairy industry were evident in participants’ ‘before’ responses, with a total of seven themes identified (Table 4). Positive associations with the dairy industry were classed as follows: (1) Dairy farming as an enterprise that entails *hard work* (n = 7 of 50 participants): i.e. top of mind responses of “*a lot of work*” _[P9]_, “*labour intensive*” _[P35]_ and “*dedicated farmers*” _[P10]_, that referenced the long hours and labour involved in dairying and often associated with respect for the farmers involved; and (2) dairy farming as an *idyllic*, *important* activity (n = 4), i.e. generalized notions of dairying as a wholesome, family-friendly pursuit with a distinct place in the rural landscape, e.g. “*good way of life*, *essential for our area*” _[P24]_, “*wholesome country*” _[P41]_, and “*enjoyable environment for family life*,” _[P13]_.

**Table 4:** Description of industry perception (IP) and FAW value themes and the percentage of participants referencing each theme before visiting the dairy farm.

Theme	Participants (n = 50)^1^	Theme description
IP (+)		
*Hard work*	7	Acknowledgement of the hard work of dairying and respect toward farmers
*Idyllic & important*	4	Agrarian views of dairying as wholesome, idyllic and positive for family and community
IP (ambiguous)		
*Dairy products*	29	Associations of dairying with its end products (e.g. milk and ice cream) and references to wholesomeness and health
*Sensory*	8	Visceral, sensory responses to dairying, e.g. references to smells of the farm
IP (-)		
*Industrial*	7	Mechanization and industrialization of dairying as harmful, particularly for animals
*Profit-oriented*	4	Prioritization of economic goals over animal welfare
*Big = bad*	3	Growth and size of dairy farms as bad for cows
FAW values		
*Biological*	36	Reference to feed and water (resources), physical health, hygiene, shelter
*functioning*		
*Natural living*	33	Reference to allowing animals to lead natural lives, e.g. pasture and/or outdoor access, space, freedom, social and individual behaviors
*Affective states*	11	Reference to animals experiencing peace, quiet, happiness, and freedom from pain, discomfort and stress
*Humane care*	28	Reference to gentle treatment and attention to individual animals; routine management duties (e.g. regular milking) and good stockmanship; avoidance of abuse
*Drugs*	11	Concerns about administration of “drugs,” e.g. antibiotics and/or hormones
*Respect for life*	5	References to end of life and short lifespan; killing of bull calves

^1^ The sum of n does not equal 50 within each category as participants often referenced multiple themes, and some participants did not reference any themes within a category.

The most predominant theme (n = 29) describing perceptions of the dairy industry referenced (3) *dairy products* (e.g. “*nice good food*” _[P41]_ and “*cheese*, *milk*, *ice cream*” _[P49]_). Arguably this theme could reflect positive associations with the dairy industry by way of the pleasure derived from consumption of its products, or alternatively, indicate that the participant had hitherto given little to no thought to dairy production. Because of this ambiguity, we did not assign a valence to this theme. Eight participants also gave responses that indicated (4) *sensory* associations with the dairy farm, most notably by referencing the “*smells*” of the farm environment. This theme was likewise not assigned a valence due to ambiguity of participant responses.

Negative associations with the dairy industry included: (5) dairy farming as *industrial* (n = 7), involving objections to “*intensification*, *industrialization*, *mechanization…killing*” _[P18]_ and to “*factory like conditions that are not humane*,” _[P11]_. Four participants also perceived dairy farming as (6) *profit-oriented* and so made objections about the prioritization of production and economic earnings over attention to animals, e.g. “*It’s a business*. *I don’t think people take the time…they are just pushing them through the turnstile*,” _[P2]_. Finally, embedded within three participants’ comments were notions that (7) *big = bad*, suggesting that larger farms were worse for animal welfare: “*large commercial farms seem to have more emphasis on production [than] animal welfare*,” _[P9]_.

#### Animal welfare-specific perceptions, concerns and values

Before visiting the farm, participants were divided in their assessment of the overall quality of life for dairy cattle: 21 respondents were at least *confident* that dairy cattle generally had a good life, but 15 were *neutral* and 14 were *not confident*.

Participants considered the following elements as necessary for dairy cattle to have a good life (in decreasing order of frequency, n = 50): fresh food and water (n = 35), pasture and/or outdoor access (n = 28, often with specific mentions of fresh air and sunshine), gentle and humane care (n = 28), space and freedom to perform behaviors (n = 24), hygiene (n = 10), shelter (n = 9), absence of stress (n = 6), social companions (n = 5), health (n = 5) and safety from predators (n = 3). These elements were organized into the following FAW value criteria (Table 4):

1. *Biological functioning* emphasized provisions for the health and physical condition of the animal (e.g. fresh food and water, shelter, hygiene, health and safety), as indicated by requirements for a good life like, “*food*, *water*, *adequate sleep*, *safe environment (no predators)*, *adequately hygienic*,” _[P33]_.
2. *Natural living* emphasized an animal’s ability to live as “*natural[ly] as possible*,”_[P44]_. Participants articulated different elements of *natural living*. These included: exposure to pasture and other outdoor elements such as fresh air and sunshine, normal social interactions with other cattle, space to carry out natural behaviors, and access to foods deemed natural. For example: “*pasture with a variety of plant life*, *some budd[ies]*, *sun and shelter*” _[P21]_.
3. *Affective states* focused on the animal’s mental well-being and included references to peace and quiet, happiness, comfort and an absence of stress and pain, e.g. “*keep their stress level down*,” _[P1]_.
4. *Humane care* emphasized the care and attention provided by humans, with participants mentioning compassionate attention at the level of the individual animal, gentle handling techniques, and consistent and predictable management. For example some felt that cows needed “*human kindness*” _[P32]_ or even “*love*” _[P35]_, while others focused on more practical aspects of management like regular milking.
5. *Drugs* condemned the overuse of antibiotics and hormones, e.g. “*adding growth hormones to increase production*” _[P11]_. While this theme surfaced mainly as a specific concern about cattle quality of life, for some participants this concern was also associated with effects on the environment and human health.
6. *Respect for life* included concerns about the culling of bull calves and the end of life and longevity of the cow, e.g. “*I feel bad that their life is shorter than a ‘natural’ lifespan*,” _[P44]_, which seemed to relate to fundamental concerns about lack of respect for animal life.

*Biological functioning* and *natural living* were the most commonly expressed FAW values, with 35 and 33 participants incorporating these values into their responses, respectively. These were followed by *humane care* (n = 28), *affective states* and *drugs* (each at n = 11), and *respect for life* (n = 5). Most participants included more than one FAW value in their responses, with a median value range of 2.5 (range: 0–5). For example, the comment “*food*, *water*, *shelter*, *regularly milked*, *space*” _[P47]_ referenced values for *biological functioning* (food, water, shelter), *natural living* (space), and *humane care* (regularly milked). Although not categorized as a FAW value, seven participants also acknowledged that FAW is variable among farms and is affected by multiple factors, e.g. “*I think it depends on the farmers’ husbandry skills*,” _[P1]_.

Not surprisingly, participants’ concerns about dairy cattle welfare typically reflected uncertainty about whether they believed these value criteria were being met on dairy farms. ‘Before’ FAW concerns thus included the following issues: (1) insufficient, biologically inappropriate, unnatural feed, (2) lack of pasture access and indoor confinement (with related concerns about overcrowding and behavioral restriction) and (3) abusive treatment of cattle. In addition, nine respondents indicated that they did *not* have any concerns about the welfare of dairy cattle, evident through responses such as, “*generally I feel that most dairy cattle would have a good quality of life*” _[P2]_ or more simply, “*don’t have any [concerns]*,” _[P5]_.

#### Knowledge and quiz scores

We did not find any relationship between self-reported knowledge and ‘before’ quiz scores. Although most participants indicated that they were either “somewhat” (n = 22) or “not” (n = 25) knowledgeable about dairy farming, ‘before’ scores on the dairy husbandry quiz were fairly high, with a median correct response rate of 3 out of 5 questions (2.9±1.1 [mean±SD], range: 1–5).

Even before touring the farm, many participants scored well on questions about basic dairy cattle feeding and housing practices: 37 of the 50 participants answered the diet question correctly, and the majority indicated that dairy cows in British Columbia were not routinely tethered in their stalls (n = 36) and that pasture access was not mandatory (n = 30). Most participants (n = 29) correctly answered that dairy cattle must give birth to a calf to give milk, but only 13 people correctly answered that calves were separated from the dam immediately after birth.

#### Relationship between demographics and ‘before’ survey responses

Sex, age, education, rural-urban status, and self-reported knowledge were not associated with FAW value expression or range, other than a relationship between age and expression of the FAW value around drugs (χ_2_ = 4.2, df = 1, p = 0.04). This relationship was driven by no respondents under the age of 34 referencing this value versus nearly half (n = 20) of participants aged 45 and older who did.

We likewise did not detect any relationship between self-reported knowledge and FAW value expression or value range. However, ‘before’ quiz scores were related to expression of the FAW value for *biological functioning* (χ_2_ = 9.2, df = 1, p = 0.024) such that participants with higher ‘before’ scores were also more likely to incorporate the *biological functioning* value into their responses (e.g. around half of participants with scores of 1 or 2 referenced this value, versus most of those scoring 3 and 4 and every person scoring 5).

We also found a relationship between ‘before’ confidence in the welfare of cattle and expression of the FAW value for *natural living* (χ_2_ = 8.20, df = 1, p = 0.0042). A lower confidence that dairy cattle had good lives was associated with expression of the value, with an overwhelming majority of non-confident and neutral participants expressing the value for *natural living* vs. less than half of confident participants.

### Responses after visiting the dairy farm

#### Perceived representativeness of the toured farm

The ‘after’ survey was generally worded to elicit participants’ perceptions regarding the farm they had just visited, but it also included a question for participants about their perceptions of conditions on the toured farm relative to other dairy farms in British Columbia. Most participants perceived conditions on the farm to be better than (n = 31) or about the same (n = 11) as other British Columbia dairy farms, with the remaining eight participants uncertain about how the farm compared to the broader dairy industry. No participant considered the observed conditions to be worse than other dairy farms in British Columbia.

#### Shifts in quiz score

**Fig 1:**
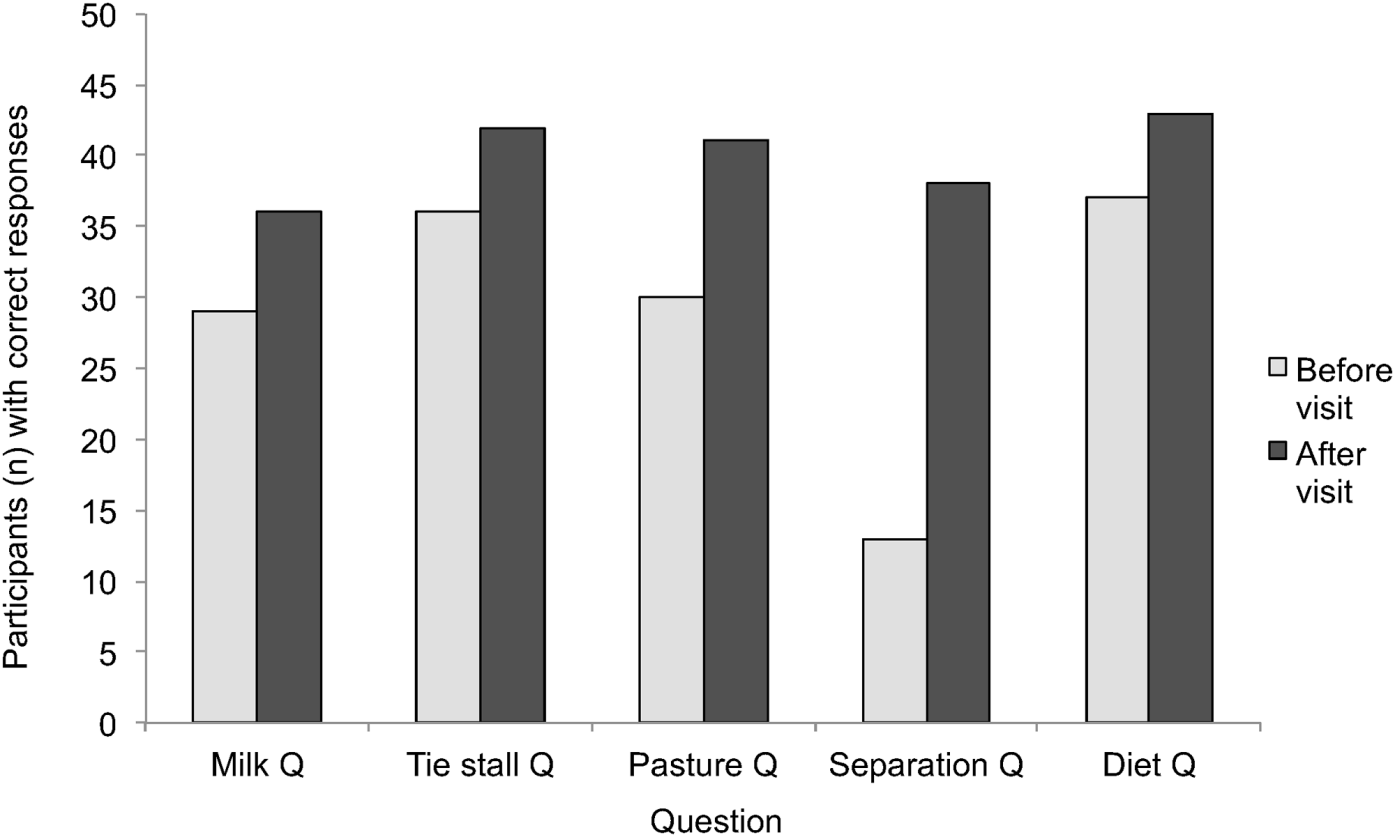
Frequency of participants with correct responses on dairy husbandry quiz questions, before and after the dairy farm visit. Milk Q = A dairy cow needs to have a calf to keep producing milk. Tie stall Q = Dairy cows in British Columbia are routinely tied to their stall in the barn. Pasture Q = All dairy cows in British Columbia are allowed access to pasture. Separation Q = How many days after birth does the dairy calf typically stay with its mom? Diet Q = Which best describes what most adult cows are typically fed on dairy farms?

Median quiz score after the farm visit was 4 out of 5 questions (4.0±0.8 mean±SD), indicating that participants answered over 1 additional question correctly after the farm visit. The frequency of participants with correct responses improved for each of the quiz questions (Fig 1). Of the 14 individuals who changed responses after the tour to the question about pasture access, 13 changed in the right direction (i.e. now responding that pasture access was not required; p<0.001). Of the eight respondents who changed their answer to if the cow needs to have a calf to keep producing milk, six changed in the right direction (i.e. now responding that calving was required; p = 0.05). Of the 27 people who changed their answer to the question about cow calf separation, 23 changed in the right direction (i.e. now responding that separation happened on the day of calving; p<0.0001). Changes in responses to the two other questions were not above the level expected by chance.

#### Shifts in perception

Participants’ qualitative responses to the ‘after’ questions of whether they thought cattle had a good life on the farm and whether they had any remaining concerns were coded into three categories (Table 5):

1. *Confident* indicates individuals (n = 14) who, after the farm visit, gave an unequivocally affirmative answer to the question of whether dairy cattle had a good life on this farm, such that only positive attributes and no concerns were mentioned. Examples included: “*It was better than I expected…I was expecting more crowding than what I saw at this farm*,” _[P17]_, “*Yes*, *plenty of food-unlimited milk for calves*, *yee haw*!” _[P20]_ and “*Great life*, *the owners/workers actually care for the animals*. *This farm is great to all of the animals*,” _[P41]_.
2. *Nuanced* indicates participants (n = 27) who mentioned both positive and negative attributes after the farm visit. Many individuals in this category suggested that the dairy cattle generally seemed to have a good life, but they also raised specific concerns, e.g. “*Fairly fair life for a cow [but] I am sure they would love to be outdoors*,” _[P29]_, “*I guess so*. *They are healthy*, *they have some freedom of movement*, *they can eat and drink as much as they want…[but] it would be nice if they could go outside more often*” _[P11]_, and “*yes [they have a good life] but I would still prefer to see animals grazing in the fields*, *eating the grass and calves not separated so quickly from mothers*,” _[P40]_.
3. *Not confident* indicates those (n = 9) who after touring the farm indicated that the dairy cattle did not have good lives. These individuals made no mention of any positive attributes. Examples included: “*Cows do not have a good life*! *Very little space to roam and are kept in unsanitary conditions …the fact that they are always indoors and standing in their own feces concerns me*,” _[P38]_ and “*No*, *conditions are poor*. *Industry is profit driven and animal welfare falls second to profit margins*,” _[P34]_.

**Table 5:** Citizens' perceptions in response to the question, "Do dairy cattle have a good quality of life?" before and after visiting the dairy farm^1^.

		**AFTER VISIT**		
		Confident (14)	Nuanced (27)	Not confident (9)
	Confident (21)	**9**	10	2
**BEFORE VISIT**	Neutral (15)	1	**10**	4
	Not confident (14)	4	7	**3**

^1^ ‘Before’ categories indicate confidence level (confident, neutral, not confident) of whether dairy cattle have good lives before visiting the farm. The ‘after’ visit category of ‘confident’ indicates participants with affirmative answers that dairy cattle had a good life on the farm with no expressed concerns; ‘nuanced’ indicates participants who mentioned concerns as well as positive attributes; and ‘not confident’ indicates participants with negative answers and no mentions of positive attributes. The bracketed numbers adjacent to or below possible response categories indicate the total participants within the respective row or column (out of 50). The number in each cell indicates the number of participants expressing each pair of perceptions before and after the farm visit. Cells outlined in bold (n = 22) indicate participants whose perceptions did not appear to shift in valence (no shift), the cells in the upper right (n = 16) indicate participants whose perceptions of the level of FAW became more negative (negative shift), and cells in the lower left (n = 12) indicate participants whose perceptions became more positive (positive shift).

#### Shifts in perceptions

We then compared these ‘after’ visit perceptions with ‘before’ confidence levels about cattle welfare to determine whether participants experienced a positive, negative, or no shift in perception relative to dairy cattle welfare (Table 5). For example, a positive perception shift would describe an individual who, before touring the farm, indicated that they were not confident about cattle welfare, but after the tour, indicated that cattle on this farm had a good life. In contrast, someone who initially expressed confidence but indicated concerns after the tour would characterize a negative shift. This approach to determining shifts in the valence of perception was designed to mitigate participant frustration in the after survey, as repetition could lead to dropout or careless responses.

We found that a minority of participants experienced a full reversal in the direction of their perceptions of cattle welfare: just two participants started confident but ended negative, and just four initially non-confident participants ended positively with no concerns. However, general shifts in perception were distributed fairly evenly among the participants such that 16 participants (approximately one-third) negatively shifted their perceptions, 12 positively shifted their perceptions (approximately one-quarter), and 22 did not shift (Table 5).

#### Concerns behind the perception shifts

There were no significant relationships between demographics and perception shift, or between ‘before’ expression of FAW values and perception shift. Thus it did not appear that expression of any particular FAW value, for example, was associated with whether or how an individual shifted their perception after touring the farm.

Qualitative analysis of the ‘after’ responses nonetheless identified some characteristics of those who shifted their perceptions positively or negatively. Those for whom the farm visit improved their perceptions of dairy cattle welfare (n = 12 of 50) cited the following attributes: the high level of care given to cattle by farm workers, plentiful food and water, a hygienic barn environment, and adequate space allotted to cattle. For example, one participant initially commented that they were concerned about “*humane treatment*, *cramped living conditions*, *and access to grazing*,” but after touring the farm remarked that they had no concerns because “*the animals seem to be well cared for…the practices on this farm seem very ethical*,” _[P45]_. Indeed, the quality of care was perceived to be positive regardless of whether participants came away from the visit with an improved perception of dairy cattle welfare overall: no participant responded that individual care toward animals was poor and many commented positively on the level of staff attention to animals.

In contrast, those for whom the farm visit negatively affected their perceptions of cattle welfare (n = 16 of 50) commented again on existing concerns or shared new concerns. The most prevalent of these new concerns was early separation of the calf from the cow, reflected in an additional 23 participants scoring correctly on the question relating to cow-calf separation after the visit. Individuals who negatively shifted their views also commented on barn space and hygiene, but in contrast to the positive shifters, negative shifters perceived the barns to be cramped and dirty rather than spacious and clean (prompting one individual to suggest, “*maybe wash the floor more often*?” _[P50]_). The most prominent complaint was the lack of pasture and outdoor access. For example, one participant who initially “*never had any concerns*” was disappointed by the farm: “*It’s not ideal…I’m sure the cows would rather be in a field…cows should have a larger freedom area*,” _[P28]_. Indeed, nearly every participant who commented on pasture or calf separation, regardless of whether that participant ultimately shifted their perception, expressed disappointment about the lack of pasture and outdoor access and surprise at cow-calf separation.

## Discussion

To our knowledge, no other study to date has examined concerns and values related to dairy cattle welfare among the North American general public, or exposed citizens to a working livestock farm and gauged their responses. This study explored how a select sample of interested citizens perceived dairy farming and its effects on animal welfare. As such, survey questions were focused on people’s ideal visions of how dairy cattle should live, thus engaging notions of how society in general should operate in relation to animal welfare in the dairy industry.

By focusing on people participating in a Slow Food^®^ tour, our sample was likely engaged in issues around food and arguably more likely to be involved as shapers of social opinion about animal welfare and other issues associated with food production. However, readers should be cautious in generalizing our results to other populations and other on-farm experiences. Rather, our approach was to provide a strong positive test of the knowledge deficit hypothesis by confronting engaged participants with one specific farm perceived to be ‘good’ by industry standards. Indeed most participants perceived the farm to have equivalent or better conditions than other dairy farms in the region. We encourage constructive replication of this study to consider other types of visitors and other farms.

### Existing FAW concerns and values

Although participants varied in their expression of FAW values in that some people’s conceptions were broader than others’ (i.e. greater range of expressed FAW values), participants collectively expressed multi-dimensional and nuanced understandings of animal welfare.

Our findings align with existing evidence that natural living tends to figure strongly in what citizens believe is necessary for farm animals to live a good life [19,21,28]. As with earlier research with non-farming citizens, participants ascribed import to: animals’ freedom to move and fulfill natural motivations [13,19,29,30], performance of natural behaviors [3,18,19,22,31], access to increased space (which is intimately connected to notions of freedom [3,21,22,31], outdoor access [3,18,19,21,31], and daylight [3,19,22]). Likewise and as with past research [21,24], our participants objected to early cow-calf separation, in part because it was perceived to be unnatural [32].

Participants also valued aspects related to biological functioning (particularly nutrition and hygiene) to the extent that this was the most frequently expressed FAW value in the current study. For example, providing dairy cattle with unrestricted access to biologically appropriate feed and clean, fresh water were among the most frequently cited requirements for a good life, a finding that again aligns with other research on lay citizen values for animal welfare [18,22,31]. As biological functioning is also highly valued by farmers and others connected to the livestock industries in Europe [13,33–35] and North America [2,36–38], these findings suggest that the values of industry and non-industry stakeholders may overlap in the area of animal health and functioning.

Regarding citizen values around affective states, most research suggests that imposing pain and other negative affective states on animals is unacceptable. For example, US surveys show that the majority believe that farm animals should be protected from pain [39] and work in Canada and the US has shown that citizens object to the performance of routine painful procedures (e.g. tail docking and dehorning) without pain relief [20,40,41]. In our study, just under one quarter of participants made direct references to affective states in dairy cattle, including happiness and an absence of pain and stress. The lack of comments may relate to our framing of the survey questions, as asking about a good life may prime responses related to elements external to the cow rather than to the cow’s internal affective state. Therefore, inferences regarding how much participants valued this aspect of FAW should be made with caution.

Interestingly, participants’ values for FAW also extended beyond the three spheres framework (biological functioning, natural living and affective states). Notably, over half of the respondents also saw the welfare of dairy cattle as intertwined with the actions and attitudes of humans charged with their care. Others have also noted the relevance of farmer-animal contact, gentle handling, and humane care to citizens’ perceptions of FAW [19,21,24]. While human actions are obviously associated with (and directly affect) biological functioning, natural living and affective states in animals, it is important to specify that the need for cattle to have attentive and loving caretakers seemed to be treated as distinct from these other three aspects.

### Effects of the farm visit

In general, the types of FAW values expressed by participants did not correlate with their performance on the knowledge-based quiz, or with their perception shifts of dairy cattle welfare after visiting the farm. The one exception to this finding was that participants with higher ‘before’ quiz scores were more likely to include biological functioning in their FAW value expression, an association which may be similar to the emphasis on biological functioning by farmers [13,31].

The ways in which perceptions, concerns and quiz performance shifted after touring the farm suggest that what participants know about animal welfare and what they value about it may be relatively independent. Hansen et al. [6] addressed how and why people’s evaluations of situations are affected by factors beyond their knowledge, and how deeply embedded values are in these processes. On the surface, it seems intuitive that familiarity breeds content and unfamiliarity, suspicion: we know, for example, that people who work within the livestock industries are more accepting of contentious practices and less concerned about animal welfare than are people unaffiliated with these industries [31]. However, emerging work suggests that learning about livestock practices fails to improve acceptance for many people, and in some cases may decrease acceptance. For example, Ryan et al. [42] found that citizens were less likely to support gestation housing for commercial sows after being exposed to various sources of information including scientific articles, images, and videos.

In the present study, performance on the quiz questions relating to basic dairy husbandry practices was variable among participants, with knowledge of some practices fairly low. For example, few respondents appeared initially knowledgeable about cow-calf separation and protocols for hormone usage in dairy cattle (the latter evident in concerns about the use of growth hormones among some participants: the relevant hormone in this case, recombinant bovine somatotropin or rBST, has never been approved for use in Canadian dairy cattle).

A key finding was that participants’ quiz performance improved after the farm visit, but this improvement was not accompanied by an improvement in perceptions of dairy cattle welfare. If we look to the divergence in confidence in cattle welfare among participants before visiting the dairy farm (21-15-14 confident-neutral-not confident, respectively) versus after the visit (12-22-16 positive-no-negative shift, respectively), it would seem that the farm visit did not result in an overall increase in confidence, as would have been predicted by the knowledge deficit model of public understanding [4,5]. Although the farm visit improved perceptions in 12 of the 50 participants, the majority experienced no shift or became more critical.

Boogaard et al. [24] wrote that, “*concerns about modern animal farming will only be allayed when information…addresses the more fundamental values that shape [public] concerns*,” (p. 281). Notably, the farm visit failed to meet the natural living criterion for most participants, particularly with regards to pasture access and cow-calf separation. Given the importance many placed on natural living, it seems that people wished to see evidence that dairy cows were kept in ways that allowed them to engage in natural behavior. In light of participants’ concerns about space allocated to cattle, future research may want to investigate whether increasing space per animal within current barn environments might mitigate public concern about zero grazing.

The problem of cow-calf separation will be more difficult to resolve. This practice illustrates how the interplay of knowledge and values inform acceptance, as this issue was not cited before people became aware of this routine practice during the tour. Early cow-calf separation is considered objectionable for a number of reasons, including concerns about the health of the cow and calf, the emotional life of both, and because it prevents both mother and calf from engaging in natural behavior [32]. One participant in the present study commented that they were relieved to see calves housed in groups and provided a high level of attention. The literature is generally consistent in showing the benefits of group housing on welfare outcomes for dairy calves [43]; something as simple as raising calves in small groups, as opposed to single housing, may help meet the concerns of those objecting to early separation out of concern for the calf, though this warrants further study.

### Considerations for further study

Past studies have estimated citizen knowledge of livestock farming through proxy questions on rural-urban background [22]. However, there is reason to question self-reports as an effective barometer of citizen knowledge: for example, though citizen participants in one study reported low familiarity with pig husbandry, many of their spontaneous welfare concerns (e.g. tail docking and teeth clipping, limited space, injured legs and joints) indeed were common issues on some pig farms [3].

To our knowledge, this study was the first attempt to gauge citizen knowledge of dairy livestock production practices through direct application of a knowledge-based quiz. We suggest that this approach provides a more accurate picture of knowledge compared with self-reports, but also acknowledge that our approach requires refinement. We did not measure confidence in answers and so cannot account for participant guessing. However, we are cautiously optimistic that the quiz yielded some useful insight into participants’ knowledge about relevant dairy husbandry practices, particularly in the case of shifts in quiz performance in a positive direction after touring the farm.

Other critiques could include our usage of the term “cows” rather than “cows and calves” in the survey, which may have biased participants to issues associated with adult animals rather than calves. Our reasoning for using the term “cows” was that in our experience, people with little cattle experience (our intended audience) use the term “cows” to refer generally to all dairy cattle, even bulls. Our results are consistent with this interpretation, as participants indicated concerns related to calves as well as cows in response to these questions.

Additionally and given the constraints associated with longer surveys (e.g. participant inattention, drop-out, frustration, [44]) we were limited in the number of questions we could ask. We made every attempt to construct quiz questions that anyone with working knowledge of dairying could answer. We also acknowledge that the present study was not designed to determine the persistence over time of any changes in knowledge, perceptions or concerns examined here. We recommend that future studies incorporate follow-up questionnaires to explore how a farm visit may influence perceptions beyond the day of the visit.

### Conclusion and implications

This study was the first to explore perceptions, knowledge, and values of animal welfare among North American citizens in the context of a farm visit. The people surveyed held nuanced conceptions of animal welfare that extended beyond those traditionally referenced in the literature. Allowing citizens to tour a dairy farm improved their performance in a knowledge-based quiz of dairy husbandry practices, but did not improve perceptions of dairy cattle welfare for most participants. Shifts in perception appeared to be primarily rooted in whether various values for animal welfare were satisfied; the tour appeared to satisfy values around humane care, but failed to meet values for natural living. The implication is that the livestock industries cannot expect one-way education efforts (even immersive experiences such a farm tour) to resolve societal concerns about animal welfare. Rather, engagement between the livestock industries and the public should be two-way such that industry stakeholders strive to hear, and respond to, concerns that result from increased transparency. This type of communication might allow industry stakeholders to better identify welfare concerns in society and to highlight shared values (such as with good animal health), providing a foundation to resolve more contentious issues.
